# One Board or Three?

**Published:** 1894-09

**Authors:** 


### ONE BOARD OR THREE?

It is certain that the next legislature will again be asked to make some law properly regulating the practice of medicine. That we have none already is a shame on the State, for which we have to thank our errant brethren, the eclectics and homeopaths, and their coadjutors, Quack-Doctor Flower and his hired lawyer, Mr. Hammond. We should also mention in this honorable connection the two physicians in the House who were *participes criminis* in the death of the bill. "What private griefs they had I know not; *they are all wise and honorable men*."

But the matter at issue will not be surrendered. We have already served notice on the congenial spirits of the opposition that the friends of protective legislation will not be satisfied until there is some statutory enactment on this subject.

We do not know in what shape the matter will next be presented to the legislature. It is our conviction that the purposes for which examining boards are established *can best be conserved by having only one board*, with proportionate representation of the different schools of medicine. This is the method now operating with success and satisfaction in New Jersey, Virginia, North Carolina, Minnesota, Colorado, Utah and Washington. There is no reason why the conjoint board should impose any hardship on those whose school is represented in the minority. On the Minnesota board there are two homeopathic physicians. One of these, speaking of the workings of his board, has said that he was "entirely satisfied with the way the remainder of the board conducted the examination, and with the fair way in which he was treated and allowed to conduct his examination. He considered the common board the best."

But judging from past experience we fear that an average Georgia legislature will not be friendly to a measure providing for a board so constituted. The compromise measure providing for three independent boards, introduced by the eclectics, was more favorably considered by the last legislature, and really lost by default. While not entirely indorsing the three board arrangement we can but regard it as infinitely better than nothing, which we have at present. The legislature may be petitioned for three boards. This ought to disarm all opposition, except, probably that of Q. Dr. Flower and his $100-lawyer. The eclectics have already declared that they would support such a petition, and promised "every means in their power to secure such legislation."

New York, Pennsylvania, Connecticut and California are now operating the three-board system, and a modification of it is in use in Maryland. The Pennsylvania law may be taken as the type of this class. It provides for three boards, regular, homeopathic and eclectic, each composed of seven members. There is also a medical council, composed of the presidents of these boards with the Lieutenant-Governor, the Attorney-General, the Secretary of State and the President of the State Board of Health. The examining boards examine the applicants and make their reports to the medical council. The licensing power is vested in the council. This law has not yet been in operation long enough for any estimate to be made of its working. We see no reason why it should not succeed in either Pennsylvania or Georgia. We believe three boards would accomplish in a great measure the objects which are sought. They would certainly keep out of our State the great army of medical Coxey-ites which are now adrift on the country.

